# A Conceptual Enterprise Framework for Managing Scientific Data Stewardship

**DOI:** 10.5334/dsj-2018-015

**Published:** 2018-06-28

**Authors:** Ge Peng, Jeffrey L. Privette, Curt Tilmes, Sky Bristol, Tom Maycock, John J. Bates, Scott Hausman, Otis Brown, Edward J. Kearns

**Affiliations:** 1 North Carolina State University, Cooperative Institute for Climate and Satellites - North Carolina (CICS-NC), US; 2 NOAA's National Centers for Environmental Information (NCEI), 151 Patton Avenue, Asheville, NC 28801, US; 3 NASA's Goddard Space Flight Center (GSFC), Greenbelt, MD 20771, US; 4 United States Geological Survey (USGS), W 6th Ave Kioling St, Lakewood, CO 80225, US; 5 John Bates Consulting, Inc., 6 Coventry Woods Drive, Arden, NC 28704, US; 6 NOAA Office of the Chief Information Officer, 151 Patton Avenue, Asheville, NC 28801, US

**Keywords:** Scientific Data Stewardship, Information Management, Enterprise Framework, Maturity Matrix, PDCA-cycle, Research Data, Open Data, Domain Stewards

## Abstract

Scientific data stewardship is an important part of long-term preservation and the use/reuse of digital research data. It is critical for ensuring trustworthiness of data, products, and services, which is important for decision-making. Recent U.S. federal government directives and scientific organization guidelines have levied specific requirements, increasing the need for a more formal approach to ensuring that stewardship activities support compliance verification and reporting. However, many science data centers lack an integrated, systematic, and holistic framework to support such efforts. The current business- and process-oriented stewardship frameworks are too costly and lengthy for most data centers to implement. They often do not explicitly address the federal stewardship requirements and/or the uniqueness of geospatial data. This work proposes a data-centric conceptual enterprise framework for managing stewardship activities, based on the philosophy behind the Plan-Do-Check-Act (PDCA) cycle, a proven industrial concept. This framework, which includes the application of maturity assessment models, allows for quantitative evaluation of how organizations manage their stewardship activities and supports informed decision-making for continual improvement towards full compliance with federal, agency, and user requirements.

## 1. Introduction

Scientific data stewardship is a fast-evolving area of research. Data stewardship has been largely considered as a subset of data management and within the scope of data governance (Plotkin 2014). Traditionally, the duties of data managers and stewards are to manage, curate, and serve data to meet user demand. For example, they are responsible for curating metadata, ensuring data fixation, and supporting data access. However, directives, recommendations, and guidelines established in the last two decades by the U.S. federal government, federal and other funding agencies, scientific organizations and societies, and scholarly publishers have greatly expanded the scope of stewardship for federally funded digital research data (Peng 2018).

Scientific data stewardship now “encompasses all activities that preserve and improve the information content, accessibility, and usability of data and metadata” (NRC 2007). Federally funded digital research data are required to be:

– preserved and secure;
– available, discoverable, and accessible;
– credible, understandable, and interoperable;
– usable and useful;
– sustainable and extendable;
– citable, traceable, and reproducible.

As a result of these expanded scopes and requirements, managing scientific data stewardship activities has exceeded the traditional responsibility and capability of most data managers and stewards. In addition to managing and serving data, they are now also responsible for contributing to features in the data, metadata, and/or points of access that enable linking between disparate datasets within or across repositories. Examples of new responsibilities include a range of activities from combining additional contextual information from other databases to the production of new integrated products, such as those for monitoring the state of the climate in the United States (Blunden and Arndt 2017) and evaluating and projecting the effects of long-term change on our natural environment and resources, human social systems, etc. (Melillo, Richmond & Yohe 2014; USGCRP 2016).

Data producers, repositories, and distributors share the responsibility of meeting these data stewardship requirements. This has increased the need for a more formal approach to managing stewardship activities in a manner that supports compliance verification and reporting. Unfortunately, many organizations, including science data centers, lack an integrated, systematic, and holistic framework that can support such effort. Such a framework also needs to provide guidance on assessing and improving both institutional stewardship capability and the quality of individual data products throughout the data product lifecycle.

Enterprise data stewardship frameworks, including those for data management and information quality management, are available to help businesses to effectively manage their data and information (e.g., Plotkin 2014; Stvilia et al. 2007; CMMI 2014). These business- and process-oriented frameworks tend to focus on moving data stewardship into a governed and managed stage based on business best practices. While it is always beneficial to improve the effectiveness and credibility of data stewardship through better-managed processes and more clearly defined procedures and guidelines, many federal data centers have just begun to address this challenge (e.g., Faundeen 2017). Business organizations are mostly managing their own data while many science data centers often need to preserve and/or provide stewardship services for other people’s data. In addition, while the quality of business data products tends to be more managed, analyzed, and well-defined, it is still fairly inconsistent or more diverse in how information on quality is curated and presented for scientific data products (Ramapriyan et al. 2017). Furthermore, utilizing a business-based data management assessment model to evaluate and improve process maturity relies heavily on a certified or authoritative entity (person, group, or institution). It could be a costly, extensive, and lengthy undertaking and may not be cost-effective or financially feasible for many science data centers at this time. In addition, such a framework may not allow data centers the flexibility of defining their own maturity requirements based on their own institutional requirements, resources, and user needs, while utilizing best practices and standards within their domains. Efforts have been underway by various Earth Science organizations and large data stewardship programs to provide guidelines and reference models to define, capture, and convey consistent data product quality information to end-users (see Ramapriyan et al. 2017 and Peng, Ramapriyan & Moroni 2016 for overviews of those efforts). So far, however, most of those effects tend to be initiated only to address project- or program-specific requirements.

Effective long-term scientific data stewardship touches on processes, standards, and best practices in multiple knowledge domains, including science, data management/preservation, and technology (Peng et al. 2016a, 2016b). Trans-disciplinary knowledge integration is required when creating procedures and guidelines for data preservation, stewardship, and use/reuse. Accurate and appropriate description of scientific data and findings, including the meaning and intended purpose of scientific data and their provenance, is vital for effective data use and decision support but requires involvement from scientific domain experts (NRC 2005). The constant advancement of data technologies and user demand for open and accessible data delivered through continuously improving methods requires the participation of technology engineers and cyberinfrastructure experts in data stewardship (Chisholm 2014; Peng et al. 2016a). Prior to defining processes and creating procedures and guidelines, comprehending the complexity of required multidisciplinary knowledge is vital. Possessing comprehensive cross-domain knowledge and expertise is necessary but extremely challenging for any single individual. Stewardship strategists and practitioners can benefit from the existence of a holistic, integrated, high-level stewardship reference framework to help understand the current state and develop improvement strategies.

Therefore, there is a need for:

– consistently developing stewardship processes and evaluating their compliance with stewardship requirements, from an organization-wide and data-centric perspective;
– consistently evaluating practices applied to individual datasets throughout their entire life cycle;
– consistently conveying quality information for enhanced transparency and interoperability and enabling trustworthiness of data and information.

In this paper, we propose a conceptual enterprise scientific data stewardship framework (ESDSF) to help organizations address this need. The term “enterprise” in this paper denotes being both organization-wide and systematic. This conceptual enterprise framework is based on the philosophy behind the proven industrial concept of the Plan-Do-Check-Act (PDCA) cycle. It provides a frame of reference and a high-level approach to effectively managing long-term scientific data stewardship activities for organizations, such as repositories, data archives, or data centers (hereinafter collectively referred to as data centers), seeking to be compliant with federal government policies and mandates, agency guidelines, organizational strategic goals, and user requirements. It aims to provide a holistic, data-centric view of managing scientific data stewardship activities and to help guide improvement at all levels of data stewardship. At the highest level (the organizational process capability level), a data-centric approach helps maintain or convey the importance of data and its high-level requirements to all entities, including management. At the lowest level (the practice level), the data-centric approach allows for consistency in defining procedures and guidelines for data development, preservation, stewardship, and services; evaluating or identifying gaps in practices applied to individual data products based on domain best practices and standards; curating and conveying quality information to users; and supporting effective system integration.

The paper is organized as follows. We first provide definition of some key terms used in this paper in Section 2. In Section 3, we introduce the conceptual enterprise framework for managing stewardship activities of geospatial data and information, adapting the PDCA cycle. The rationale and basic description of each stage of the adapted PDCA cycle is described. Summary and discussion including limitation and potential application of the enterprise framework are provided in Section 4.

## 2. Definition of Key Terms

Generally speaking, data can refer to anything that is collected, observed, or derived and used as a basis for reasoning, discussion, or calculation. Data can be either structured or unstructured, and can be represented in quantitative, qualitative, or physical forms. Scientific or research data is defined as: “the recorded factual material commonly accepted in the scientific community as necessary to validate research findings” (OMB 1999).

In this paper, a *data product* refers to “a product that facilitates an end goal through the use of data,” usually with a well-thought-out algorithm or approach (Patil 2012). Data products tend to be structured and can be raw measurements or scientific products derived from raw measurements or other products. Products can also be statistical or numerical model outputs, including analyses, reanalyses, predictions, or projections. Earth Science data products may be further categorized based on their processing levels (e.g., FGDC 2002).

A *dataset* is an identifiable collection of physical records, a digital rendition of factual materials, or a product of a given version of an algorithm/model. A dataset may contain one to many physical samples or data files in an identical format, having the same geophysical variable(s) and product specification(s), such as the geospatial location or spatial grid. Data products in this paper are limited to digital geospatial data products and we may use dataset and data product interchangeably.

A *research finding* is an intellectual opinion expressed by an entity (person, group, or institution) based on examination or analysis of data, representing the current knowledge on a certain topic or subject. It may be available publicly in the form of scientific reports, journal articles, images, or videos.

Although it is often used interchangeably with research finding, a *scientific finding* in this paper refers to a statement summarizing what an entity (person, group, or institution) found when reviewing the whole corpus of scientific research in a given area.

*Information* is considered as data being processed, organized, structured, or presented in a given context, while *knowledge* is gained from an understanding of the significance of information (Mosely et al. 2009; Ramapriyan et al. 2002). Data and information may overlap and may be used interchangeably.

**Figure 1:**
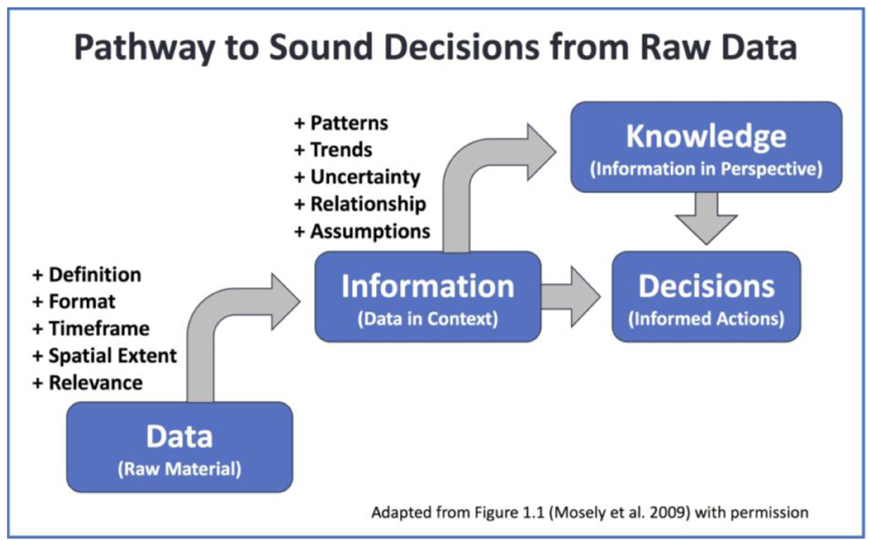
Diagram of pathway from raw material to informed decisions, adapted from Figure 1.1 of Mosely et al. (2009) with permission.

Geospatial data products organize and provide physical, spatial, and temporal relevance to raw digital measurements or physical samples. Research findings uncover patterns and relationships, which help further our understanding. They may also derive and present quality information for specific data products. Scientific findings help summarize state-of-the-art information and capture expert knowledge. Both information and knowledge contribute to informed decision-making (Figure 1).

*Provenance* is information about the origin and history of entities, activities, and people involved in producing a piece of data or thing. Provenance can be used to form assessments about its quality, reliability or trustworthiness (Tilmes et al. 2015a). Provenance is important for both data products and scientific findings (e.g., Hills et al. 2015; Tilmes et al. 2013; Ramapriyan et al. 2016).

A *maturity model* refers to a maturity reference or assessment model with desired evolution in discrete stages from a certain aspect or perspective of scientific data stewardship.

In systems engineering and requirements engineering, *a non-functional requirement* (NFR) is a requirement that specifies criteria that can be used to judge the operation of a system, rather than specific behaviors (Chung and do Prado Leite 2009). NFRs are required characteristics of a system, often called qualities of the system. Other terms include “quality attributes”, or “constraints”. For example, the NFRs associated with the FAIR data principles defined by Wilkinson et al. (2016) are findability, accessibility, interoperability, and reusability.

*Functional requirements (FRs)*, conversely, are about functions, activities, tasks, etc., that may accept some input and produce some measurable output (Chung 2013). For example, the FRs associated with accessibility of the FAIR principles include: i) (meta)data are retrievable by their identifier using a standardized communications protocol and ii) metadata are accessible, even when the data are no longer available (based on Wilkinson et al. 2016).

NFRs in this paper refer to those constraints imposed by the federal government directives on stewardship for federally funded digital scientific data as well as those by users. Table 1 provides a list of NFRs that can be derived from current federal requirements. FRs in this paper refer to functions, activities, and tasks that need to be carried out to be compliant with the defined NFRs.

## 3. Enterprise Scientific Data Stewardship Framework

In this paper, scientific data stewardship is considered and addressed from two distinct perspectives. One is Scientific Data-Stewardship: that is, a systematic, scientific way of stewarding data. The other is Scientific-Data Stewardship: that is, the stewardship of scientific data. Stewardship of scientific data requires not only ensuring the integrity and accessibility of data but also its meaning and usefulness. Ensuring and improving the content and value of the scientific data is a big part of Scientific-Data Stewardship.

**Figure 2:**
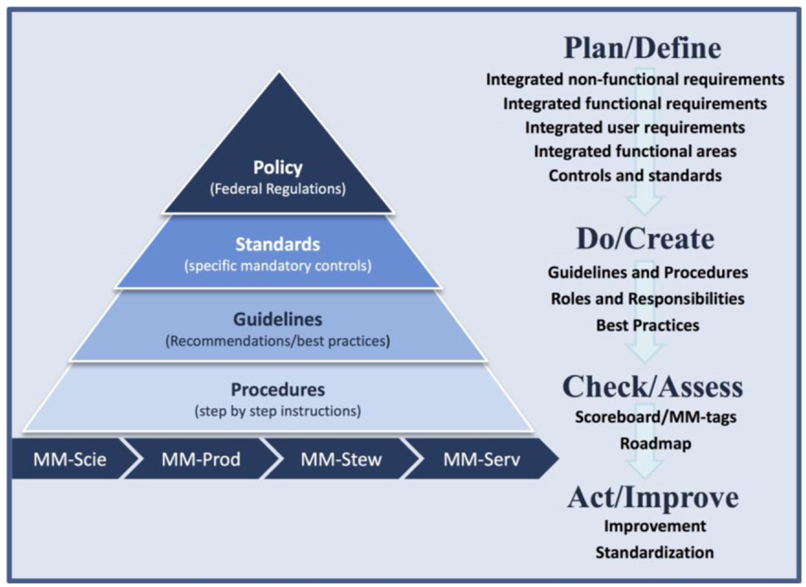
Conceptual diagram of proposed data-centric, enterprise scientific data stewardship framework. The staggered pyramid on the left represents interconnection between federal regulations, mandatory controls, recommendations, and instructions. The MM-tags beneath the pyramid represent quality assessments through the entire data product life cycle. The text on the right represents each step of the PDCA cycle and a summary of high-level outcomes

The enterprise framework described in this paper, as outlined in Figure 2 and Table 2, follows the philosophy behind the Deming or Shewhart cycle of Plan-Do-Check-Act (PDCA). The PDCA cycle is a proven, iterative four-step management model in business and industry for the control and continual improvement of processes and products (Shewhart 1939; Deming 1986). Shewhart (1939) described a three-step model for statistically controlling the quality of a manufactured product: specification, production, and inspection. Deming (1986) extended the Shewhart model into a four-step model, adding explicitly the dimension of being progressive and cyclical. The PDCA cycle is commonly used in process improvement and quality management both for individual products or organizations. It focuses on measured, progressive, and continued quality improvements of a process or product to achieve the desired outcomes. As described by Nayab and Richter (2013), the flow diagram of the PDCA model presents the concept of “planning the required changes (plan), making the changes (do), checking whether the implemented changes have the desired effect (check or study), and institutionalizing the changes (act).”

In this paper, we adapt the PDCA cycle as a flow diagram to describe a high-level, holistic, and integrated approach for effectively managing scientific data stewardship activities. We use the philosophy behind the PDCA cycle to present the concept of defining integrated requirements (plan/define), creating procedures and guidelines (do/create), assessing the current maturity of processes and practices (check/assess), and taking steps to improve or institutionalize the processes and procedures for meeting these requirements (act/improve). For clarity and for demonstration purposes, both the conventional use and how we intend to adapt and reuse the PDCA cycle are outlined in Table 2.

Often, it is necessary to first establish a baseline and to identify potential gaps for an organization in its currently defined data stewardship processes, procedures, and practices. This is similar to the observing stage in a different version of the PDCA cycle, i.e., OPDCA. This helps the organization understand where it is and where it needs to be. Based on available resources, it can make an appropriate plan to take incremental and informed steps towards stewardship improvement.

In addition to being data-centric, this proposed ESDSF aims to treat scientific data stewardship as a whole with a vertically integrated (policies, processes, procedures, practices) and horizontally integrated (data product development, production, preservation, services) approach. The following subsections provide rationales and goals for each step of the PDCA cycle.

### 3.1. Plan/Define

To complete any task correctly in a timely fashion, one needs to know what to do, how to do it, and how to do it right. Good planning is fundamental to the success of any project or undertaking. It is the same for data centers seeking to effectively fulfill their obligations in managing and stewarding federally funded scientific data.

U.S. federal directives, agency guidelines, and organizational strategies impose high-level constraints and requirements on data centers or services. They are sometimes called “mission parameters” as they define and constrain agency missions. Defining and keeping up to date with the NFRs is necessary to ensure compliance and effectively manage their stewardship activities, while FRs need to be integrated and consistent with NFRs to minimize the chance of potential gaps in developing processes and creating procedures.

Policies and mandates may be issued for various aspects of developing, producing, preserving, analyzing, and servicing federally funded research data. These directives and administrative policies at the highest level of the Executive Branch in the United States provide the context and specific guidelines for specific agency and data center planning. Thorough and regular review of relevant federal policies and mandates and agency guidelines will help ensure that the defined NFRs are integrated and up-to-date—this can serve as a first solid step towards ensuring agencies’ compliance. Examples of NFRs that can be derived from the federal directives on federally funded scientific data can be found in Table 1.

The needs and requirements of data users may change over time. New users or applications may create new data requirements; one cannot service the diverse needs of data users if one doesn’t know what they are. Therefore, it is important to interactively communicate with the current and future data users, such as through effective user engagement activities. The user engagement strategy of NOAA’s National Centers for Environmental Information (NCEI) outlines some best practices (Arndt and Brewer 2016). At the same time, it is usually not practical (and may not have the best return on investment) for data centers or service providers to cater to each user’s individual needs and requirements, except in cases with a high impact for society or organizational strategy. Sometimes, individual requirements may be supported by specific internal or external funding resources. Commercial entities may be better positioned to offer more tailored solutions. For data centers, the key to best utilizing resources is to actively engage with user communities to understand the range of data-use needs and to identify and implement items that will best address those needs, both for a specific user domain (e.g., Kruk et al. 2017) and those of the whole data user community (e.g., Brewer, Hollingshead & Owen 2017).

Communicating these defined NFRs and FRs across the entire organization will help set a clear direction, minimize redundancy in implementation, and maximize efforts to achieve the goal of effectively managing data stewardship activities. Compared to the conventional PDCA cycle, this plan/define stage is less evolving because the requirements are integrated and holistic, so the progressive and continual improvement tend to happen at the next three stages rather than in this first stage. Still, the requirements are not intended to be static, and periodic review is necessary.

### 3.2. Do/Create

Once integrated functional and nonfunctional requirements and user needs are defined or identified, corresponding procedures and guidelines will need to be created to guide the actual operation of data centers. Many federal agencies have created agency-level principles, procedure directives, and guidelines to help their line offices to be compliant with federal directives. See Appendix A for examples from selected U.S. Earth Science agencies and international organizations. If agency-level procedure directives and guidelines are not available, individual institutions or line offices will need to create their own procedures and provide guidelines to people who implement the procedures.

**Figure 3:**
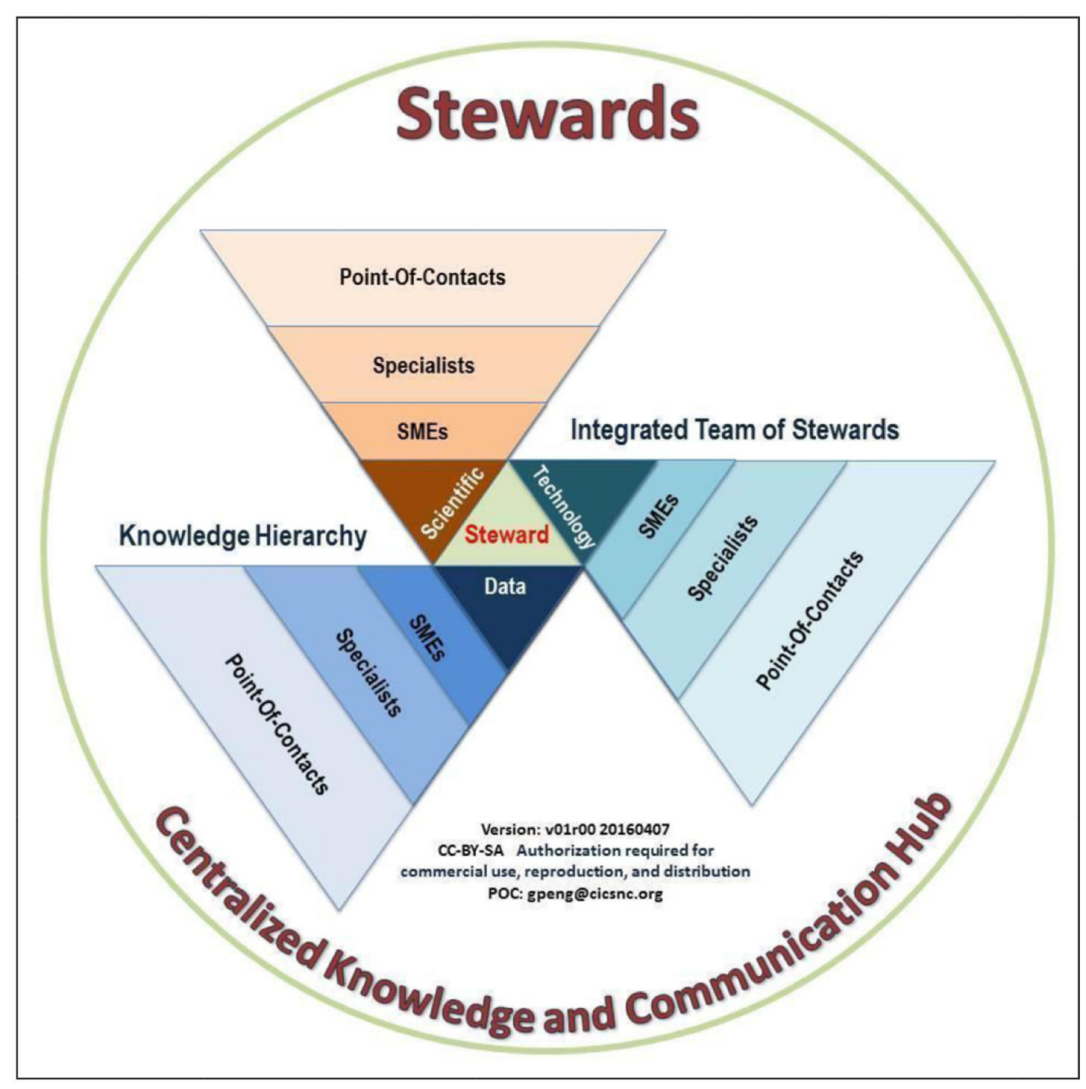
Diagram of an integrated team of stewards from multiple fields serving as a centralized knowledge and communication hub for effective long-term scientific data stewardship. SMEs denote domain subject matter experts. The concept of this diagram is based on Peng et al. (2016a)

Implementing these agency-wide procedures and doing it well requires leveraging domain best practices. Although the development of the institutional best practices is sometimes limited to the expertise and knowledge of individual persons or organizations, community standards and best practices are proven through broader and more diverse experiences of multiple organizations and research to achieve the desired results (TechTarget 2007). Identifying community standards and best practices requires both domain knowledge and gained experience. The knowledge and capability hierarchy in Figure 3 depicts both the experience-gained path and a number of people in each category within an organization (see Table 3 for definitions of the roles, required knowledge, and their responsibility or required minimum capability). Developing these roles and responsibilities helps organizations to better manage their human resources and facilitate the implementation of developed processes and procedures.

Effective communication across domains is essential because scientific data stewardship touches on multiple knowledge domains. Figure 3 depicts the need for and importance of an integrated team of stewards who represent data management, technology, and science knowledge domains at the institutional level. These stewards are domain subject matter experts (SMEs) with an ethos of continuously improving institutional data assets that were initially produced by others (Table 3). They should also have a general knowledge of other domains. Generally speaking, data stewards focus on managing both datasets and metadata, technology stewards are responsible for managing tools and systems, and scientific stewards help ensure and improve data product quality and usability. Stewards will help organizations identify community best practices and standards, define procedures, and provide guidelines and oversight.

The concept of an integrated product team (IPT), including roles, knowledge input, and capability required/provided, can be adopted for individual projects for developing or acquiring products after the processes and procedures are defined. For example, a point-of-contact (POC) may be included in the IPT if limited subject knowledge input is required. A SME may serve in the capacity of a POC if there is limited time availability for the SME.

This IPT approach is not new and has been proven effective in both business and scientific communities. NOAA’s climate data records (CDR) program has adapted this approach in its research-to-operation (R2O) transition process (CDRP 2014). A CDR R2O IPT normally consists of a transition manager, a product SME/POC, and specialists from each of archive, operation, and access. The IPT works closely with the data producer or product lead who could be either internal or external. Having an IPT with clearly defined roles and responsibilities has improved the efficiency of both the transition process and human resource management.

Management support is essential for successful implementation of created procedures and best practices. Large data centers may find it necessary to have an enterprise data steward to lead, coordinate, and oversee the day-to-day stewardship effort and to implement mechanisms such as a data stewardship council to provide the governance and leadership role (Plotkin 2014).

### 3.3. Check/Assess

Before taking steps to improve, organizations need to know where they are and where they should go. Thus, informed decisions and actionable steps for improvement require assessing—preferably in a consistent and systematic way—how well the organization is doing. Having quantifiable measurements of the outcomes is one of the key characteristics of the PDCA cycle. Statistical analysis results are often used in ensuring and managing product quality (Shewhart 1939; Deming 1986). In the traditional use of the PDCA method, the ‘checking’ step is taken to measure the performance of the implementation of changes defined in the planning phase. In contrast, we propose to check how well an organization does currently against community best practices, utilizing consistent maturity assessment models. This helps identify potential gaps in processes, procedures, and practices and allows for more consistent assessment results and improvement guidance.

One of the biggest challenges in assessing quality of data and information is the fact that quality is multidimensional and multi-perspective (Lee et al. 2002; Hu et al. 2005). Based on how data consumers ranked the importance of 179 data quality attributes, Wang and Strong (1996) selected the top 15 and categorized these attributes into four dimensions: Intrinsic (accuracy, objectivity, believability, reputation), Contextual (relevance, value-added, timeliness, completeness, appropriate amount of data), Representational (interpretability, ease of understanding, concise representation and representational consistency), and Accessibility (accessibility, access security). Although data quality and information quality have been frequently used interchangeably in the literature, Hu et al. (2005) has argued that it is important for Information System professionals and organizations to distinguish information quality from data quality. They have defined data quality as the intrinsic quality of data itself and information quality as “the degree to which the information is represented and to which the information can be perceived and accessed.” Hu et al. (2005) have also pointed out that high data quality is a necessary but not sufficient condition for high information quality.

For individual geospatial data products, it is important to ensure and improve both data and information quality. Ramapriyan et al. (2017) defined four dimensions of information quality: Science, Product, Stewardship, and Service. These dimensions are based on the different activities involved in the four phases of the data product life cycle: “1. define, develop, and validate; 2. produce, assess, and deliver (to an archive or data distributor); 3. maintain, preserve and disseminate; and 4. enable data use, provide data services and user support.”

Mapping of NFRs to these two different quality dimension categorizations can be found in Table 1. Some of the NFRs, denoted by “N/A” in Table 1 including Preservability and Sustainability, are not explicitly addressed in Wang and Strong (1996)’s categorization because they focused on the quality attributes that are more important to data consumers. It is also noted that many of these NFRs, such as Representativeness and Interoperability, could impact activities in more than one phase of the data product life cycle. This is because concise representation and representational consistency for data, format, metadata, and product descriptive documents are contributing towards Representativeness and Interoperability.

For science data centers, maturity assessment models, such as those outlined in Peng (2018), could be used to consistently and quantitatively evaluate maturity of organizational processes and systems, procedures, practices, and products. These quantitative results can be captured in metadata records or quality descriptive information documents for both machine and human end users. We recommend the consistent use of maturity metadata tags (MM-tags) for improved integration to and interoperability with other tools and systems, such as catalog services. Figure 2 denotes the use of the metadata tags of MM-Scie, MM-Prod, MM-Stew, and MM-Serv to represent the data product maturity results for each of the four dimensions defined by Ramapriyan et al. (2017). This emphasizes the importance of the quality information from the entire data product lifecycle.

As indicated previously, this check/measure stage may be the first step for an organization adopting the PDCA approach to establish a baseline of its current data stewardship activities. Once the baseline and the end goal are established, the organization can then identify the highest priority steps and make informed plans for incremental improvements.

In order for decision makers to trust the data and the scientific findings resulting from analysis of this data, and to help them make informed decisions, it is essential to establish and demonstrate in a consistent and transparent way the credibility of not only the data but also the whole process of producing, managing, stewarding, analyzing, and servicing the data (Tilmes et al. 2015b). Moreover, practices within data centers to enhance data holdings with additional value-added metadata and attribution can create usable information sources for decision making.

Even when utilizing the same assessment model, the quality of the resultant maturity assessments can be influenced by multiple factors, including the experience of the entity who carried out the assessment. While having a certified or authoritative entity (person, group, or institution) carry out the assessment could better ensure the quality of the assessment results and recommendations based on the assessment, this could be a costly and lengthy undertaking that may not be cost-effective for all organizations or individual data products. In some cases, self-assessment may be carried out by a non-certified person for internal or personal use.

To help consistently capture and convey this information to users, we have defined five categories of assessments as shown in Table 4.

Self-evaluation utilizing a reference framework, such as a matrix, checklist, or an international standard, may be carried out internally by an individual person, group, or institution, with or without a formal internal review process. If the internal evaluation results are used for a certification purpose, it is recommended to make the information about the reference framework, evaluation workflow, review process, and evaluation results publicly available, along with the self-issued certificate. This not only helps meet the transparency requirement but also helps establish the credibility of the certificate. To help the public understand the nature and maturity of the certificate, one could use the assessment categories described here to convey the relevance of the certificate.

A certified assessment represents the most formal—and usually the highest quality—of the assessment results and recommendations. Achieving the highest maturity levels defined in a maturity assessment model and being accredited by a certified entity indicate that an organization or data product has achieved the highest standard of quality—a “gold seal” of approval. Such a certification underlines the completeness of well-established and well-accepted processes, standards, and practices. It helps establish the trustworthiness of individual data centers or datasets by ensuring the quality of all processes in the organization or all practices applied to the dataset, relative to the defined community standards and best practices. However, certification requires an established authoritative body. The process for establishing standards and certification processes and for establishing an accreditation body for certification can be lengthy and extensive. The formal assessment certification process is most likely to be lengthy, extensive, and costly. At the current stage, there may be a need to certify a limited number of extremely high-impact data centers or individual datasets that are crucial to the livelihood of organizations and their operations or critical to meeting organizational missions or goals, in both private and public sectors.

For many data centers or datasets, it may not be financially practical to have the assessment done and certified by an authoritative entity. However, carrying out an assessment with a reference maturity assessment model, either for a self-evaluation or as a part of an internal stewardship process, is usually less financially constrained. It will still help identify potential gaps in organization capability or practices or establish the credibility of the underlying processes or practices. As mentioned previously, the availability of the detailed justifications along with information about the assessment model will help demonstrate the capability maturity of individual data centers or practice maturity of individual datasets. This will provide a range of options to users and enable them to make informed decisions based on their own diverse use needs. For example, for time-sensitive users, such as weather forecasters, a short data latency is critical. They may need to use a product with a short data latency even if the quality of the product or the capability of the organization that provides the product may not be the best. On the other hand, climate modelers may need to use a product that is consistent over the long-term. If a data product is to be integrated into daily operations, an organization may consider availability of the data and stability of data services by data centers as the most important factors.

We believe this multi-layer and multi-dimensional matrix approach is more practical. It fills the void between the current state and the gold-seal-approval state. It offers the flexibility of allowing data centers or users to define their own process capability and data product maturity levels. It enables a more progressive, structured, and iterated improvement process. Being transparent will allow users to gain insightful knowledge about the maturity of data centers/datasets and help establish the credibility of data centers/data products.

### 3.4. Act/Improve

The quantitative information from the Check/Assess step, together with feedback from dataset users, will help organizations or individuals make informed improvement decisions and guide them in addressing improvements that are high priority to them and their users. The improvement may come in the form of adjusting current processes, procedures, and practices. If a desired maturity is achieved for the requirements, the action will be to institutionalize them. Then, a monitoring process should be in place to trigger a new PDCA cycle if a new requirement or a new area of improvement has been identified.

It has long been recognized that both data management and process management are important. If the data were not well-defined, the data could be misused in applications. If the process were not well-defined, it would be impossible to meet user’s needs. The same can be said about practices and processes of managing scientific data stewardship activities. Being both holistic and progressive helps organizations prioritize and make effective improvement decisions to ensure the success of the improvements. In practice, an organization may need to focus on improving just one or a few quality attributes at a time, such as transparency or interoperability.

Following lean practices for organizations (Poppendieck 2002) in identifying, defining, and executing improvement actions—taking small, incremental steps toward improvement—will allow organizations to quickly cycle back into assessment, determine the efficacy of the change, and make further adjustments. For instance, feedback indicating user difficulty in selecting the appropriate data for analytical uses might result in a small number of additional quality assessment annotations or additions to a product descriptive document; these changes can then be immediately tested with user groups.

Deeper-level changes such as those impacting hiring decisions and data center staff development may be indicated through organization capability maturity assessments. Generally speaking, clearly identified roles and responsibilities, such as the stewardship roles in Table 3, can help institutions effectively manage their human resources. They are also essential for effectively creating and implementing procedures.

## 4. Summary and Discussion

Data stewardship is never really a “one and done” issue. Stewardship requirements, technologies, and user demands continue to evolve, and we need a framework that can anticipate these changes and set our data systems up to meet them. Organizational excellence in the long-term stewardship of scientific data requires an enterprise-level, data-centric, holistic, and integrated approach to effectively managing stewardship activities. Such an approach should include:

- Defining, creating, continuously reviewing, and updating the following:
  - NFRs set forth by governmental directives and by end user needs,
  - FRs to be compliant with federal directives and aligned with agency guidelines and institutional strategy,
  - processes and procedures,
  - standards and best practices, and
  - roles and high-level responsibilities.
- Continuously evaluating and assessing organizational process capability and practices applied to individual data products.
- Continuously improving the quality of individual digital scientific data products and services through the entire data product lifecyle.

The important parts of this ESDSF are the quantitative evaluations and the progressive approach—taking small, informed, incremental steps toward continual improvement.

The challenge of bridging policies and implementations across an entire organization always exists. However, this is particularly challenging for scientific data stewardship because the requirements and best practices are fast-evolving, and multi-disciplinary expertise is essential. Significant progress has been made by science data centers on various aspects of improving scientific data stewardship, but many of the challenges for managing scientific data stewardship activities remain. Some data centers, large stewardship programs, or services may already have most of the individual components in place, but these components may not be as integrated or thoroughly evaluated as they should be. Organizations could adapt ESDSF to help better integrate and manage their stewardship activities to meet their own unique data stewardship needs. This work is intended to be a first step, not the last, towards a community-consensus enterprise framework for effectively managing scientific data stewardship.

ESDSF is a flexible and generalized framework that includes but does not explicitly refer to many important aspects of scientific data stewardship. A selected number of the aspects are discussed below in no particular order.

The governance of data stewardship activities is important in ensuring effective scientific data stewardship in individual organizations. For a more managed and formal approach to data governance, one can turn to an actionable guide by Plotkin (2014). For organizations without a data stewardship program, we recommend that they at least establish an enterprise steward role to oversee and facilitate the organization-wide data stewardship activities.

An important part of scientific data stewardship is capturing and documenting what has been done to a dataset, as this information is critical to data provenance. Documenting this information in a consistent and systematic way and making the information readily available and understandable to both human and machine end-users, allows for enhanced transparency, usability, interoperability, and user feedback. Optimally, effective scientific data stewardship can help establish the credibility of data and information, which is extremely important for scientific findings, as well as help enable informed and traceable decision-making.

The health and sustainability of observing networks is critical to the continuous update and extension of data products. Sustainability of observing networks includes continuity and maintenance of existing critical ground-based and remotely sensed observation systems, minimization of data gaps (in both spatial and temporal coverage), near-real-time monitoring of instrument performance, and other measures. These are probably the responsibilities of the scientific community, programs, or agencies rather than those of data centers. However, data centers could play an important role in providing much needed guidance on preservation, stewardship, and services.

The security of data and information systems, such as data production systems, ingest/storage/staging systems, and service systems, is also very critical to the trustworthiness of data products and scientific findings. The processes, procedures, security controls and best practices should be defined and implemented as an integral part of scientific data stewardship.

Another growing concern with the increasing capability of web-enabled tools and platforms is that powerful online transformations are producing ephemeral products such as images, maps, graphs, etc. that sometimes fail to link directly to the canonical stewarded data products from which they are derived. Use of such products in scientific research threatens the credibility of the results. Properly constructed tools based on, and taking advantage of, a formal holistic stewardship framework could help mitigate those concerns. With more players in the field, the challenge of data accountability becomes critical. Defining roles and responsibilities in every level of stewardship and every stage of the data product lifecycle will help facilitate this challenge.

We are in a new era of data archiving/sharing: coexistence of federal data centers and services, academic and non-profit organization repositories and services, and commercial cloud and analytics solutions. For any of these organizations, long-term health and sustainability, integrity, authenticity, and trustworthiness of data and the institution that hosts the data remain as important challenges for data preservation and data sharing. Funding agencies need to understand the importance of funding and staff sustainability for successful scientific data stewardship. Individual data centers need to understand the need for and importance of improving the skillsets of their staff to help them keep up with evolving job requirements.

## Supplementary Material

Appendix A

## Figures and Tables

**Table 1:** Examples of NFRs on federally funded digital scientific data and our mapping to information quality dimensions defined by Lee et al. (2002) and Ramapriyan et al. (2017).

NFRs	Description	Dimension based on Lee et al. (2002)	Dimension based on Ramapriyan et al. (2017)

Accessibility	The quality or fact of being accessible	Accessibility	Stewardship; Service
Accuracy	The quality or fact of being correct	Intrinsic	Science
Availability	The quality or fact of being available	Accessibility	Product
Completeness	The quality or fact of being complete	Contextual	Product
Findability	The quality or fact of being findable	N/A	Stewardship; Service
Integrity	The quality or fact of being intact	Intrinsic	Product; Stewardship; Service
interoperability	The quality or fact of being interoperable	Representational	Product; Stewardship; Service
Objectivity	The quality or fact of being objective	Intrinsic	Science
Preservability	The quality or fact of being preservable	N/A	Stewardship
Reproducibility	The quality of fact of being reproducible	N/A	Product; Stewardship
Representativeness	The quality of fact of being representational	Representational	Product; Stewardship
Security	The quality or fact of being secure	Accessibility	Stewardship; Service
Sustainability	The quality or fact of being sustainable	N/A	Product; Stewardship; Service
Timeliness	The quality or fact of being done at a useful time	Contextual	Product; Service
Traceability	The quality or fact of being traceable	N/A	Product; Stewardship; Service
Transparency	The quality or fact of being transparent	N/A	Product; Stewardship
Usability	The quality or fact of being easy to understand and use; being usable	Representational	Product; Stewardship; Service
Utility	The quality or fact of being utilized	Intrinsic	Product

**Table 2:** A summary of the PDCS cycle as defined by Nayab and Richter (2013) and adapted by ESDSF.

The PDCA Cycle based on Nayab and Richter (2013)	The PDCA Cycle adapted by ESDSF

Plan/Define *(planning the required changes)*	Integrated non-functional requirements from federal directives, agency policies, organizational strategy, and user requirements (referred to as the requirements) are defined and documented. (They may be referred to “mission parameters".)
	Functional areas, controls, and standards necessary for compliance with the requirements are defined and documented. (They are required changes.)
	They are communicated within the organization across different entities.

Do/Create *(making the changes)*	The guidelines, processes, procedures, and best practices to enable the compliance with the requirements are created, documented, and implemented.
	They are communicated within the organization across different entities to ensure consistency and efficiency.

Check/Assess *(checking whether the implemented changes have the desired effect)*	Check the results of implementations of processes and procedures using consistent assessment models that are based on community best practices, yielding quantifiable evaluation results.
	The results are captured and presented in ways suitable to both human and machine end-users.
	Areas for improvement are identified with a roadmap forward based on where they are and where they need to be.

Act/Improve *(adjusting or institutionalizing the changes)*	Steps are taken based on the roadmap forward to improve current processes, procedures, and practices, circling back to the Do/Create stage if necessary.
	If the requirements need to be updated, circle back to the Plan/Define stage.
	The processes, procedures, and practices of implementations are standardized within the organization once a desired maturity for the requirements is achieved. Monitoring is in place to trigger a new PDCA improvement cycle if a new requirement or a new area of improvement has been identified.

**Table 3:** Roles, Knowledge, and Capability: Provided or Required (with input from Chisholm 2014).

Role	Minimum Knowledge Required	Minimum Responsibility or Capability Provided

**Point-Of-Contact (POC)**	Basic, very limited knowledge in a particular subject	Serving as a focal point of information concerning an activity or program; limited knowledge input
**Specialist**	Highly skilled with extensive knowledge in a particular subject	POC + good subject knowledge input
**Subject Matter Expert (SME)**	Extensive knowledge and expertise in a specific domain	POC + extensive subject or domain knowledge input
**Steward**	Extensive knowledge and expertise in a specific domain and general knowledge in other relevant domains, e.g., science/business and technology.	SME + effective trans-disciplinary communication + mindset of caring and improving other’s assets + prompting for good stewardship practices

**Table 4:** Maturity Assessment Categories and Descriptions.

Category Number	Description

Category 1	No assessment done.
Category 2	Self-assessment—preliminary evaluation carried out by an individual for internal or personal use; abiding to non-disclosure agreement.
Category 3	Internal assessment—complete evaluation carried out by an individual non certified entity (person, group, or institution) and reviewed internally with the assessment results (ratings and justifications) publicly available for transparency.
Category 4	Independent assessment—Category 3 + reviewed by an independent entity, that has expertise in the maturity model utilized for the evaluation.
Category 5	Certified assessment—Category 4 + reviewed and certified by an established authoritative entity. Maturity update frequency is defined and implemented.
