# Supplementary material for: A Conceptual Enterprise Framework for Managing Scientific Data Stewardship
Source: Data Sci J. Author manuscript; Available in PMC 2020 Oct 22. (PMC7580807; doi:10.5334/dsj-2018-015)
Supplement: Appendix A [file NIHMS1530236-supplement-Appendix_A.pdf]

APPENDIX A

Links to data sharing plans, principles or procedure directives, and guidelines created by selected U.S. Earth Science agencies and international organizations are provided in this section.

- ❑ Agency Plans for Increasing Public Access to Research Results/Scholarly Publications
  - NOAA: https://docs.lib.noaa.gov/noaa_documents/NOAA_Research_Council/NOAA_PARR_Plan_v5.04.pdf (Version: v5.04 February 2015 doi:10.7289/V5F47M2H)
  - NASA: https://www.nasa.gov/sites/default/files/atoms/files/206985_2015_nasa_plan-for-web.pdf (Version: December 2015)
  - USGS: https://www2.usgs.gov/quality_integrity/open_access/downloads/USGS-PublicAccessPlan-APPROVED-v1.03.pdf (Version: v1.03 3/9/3016)
- ❑ Data Sharing Principles or Procedure Directives
  - NOAA: https://nosc.noaa.gov/EDMC/documents/Data_Sharing_Directive_v3.0.pdf (Version: v3.0)
  - NASA: https://science.nasa.gov/earth-science/earth-science-data/data-information-policy
  - USGS: https://www2.usgs.gov/datamanagement/share.php
  - GEO (Group on Earth Observations): http://www.earthobservations.org/dswg.php
  - WDS (World Data System): https://www.icsu-wds.org/files/WDS_Data_Sharing_Principles_2015.pdf (Version: November 2015, v1. doi:10.5281/zenodo.34354)
- ❑ Data Management Plan, Principles or Procedure Directive
  - NOAA: https://nosc.noaa.gov/EDMC/documents/EDMC-PD-DMP-2.0.1.pdf (Version: 2.0.1)
  - NASA: https://www.nasa.gov/open/researchaccess/data-mgmt
  - USGS: https://www2.usgs.gov/usgs-manual/500/502-9.html
  - GEO: https://www.earthobservations.org/documents/dswg/201504_data_management_principles_long_final.pdf
